# The Comparable Microenvironment Shared by Colorectal Adenoma and Carcinoma: An Evidence of Stromal Proteomics

**DOI:** 10.3389/fonc.2022.848782

**Published:** 2022-04-01

**Authors:** Keqiang Yan, Bin Bai, Yan Ren, Benliang Cheng, Xia Zhang, Haichao Zhou, Yuting Liang, Lingyun Chen, Jin Zi, Qinghai Yang, Qingchuan Zhao, Siqi Liu

**Affiliations:** ^1^ College of Life Science, University of Chinese Academy of Sciences, Beijing, China; ^2^ Department of Proteomics, Beijing Genomics Instituion (BGI)-Shenzhen, Shenzhen, China; ^3^ State Key Laboratory of Cancer Biology & Department of Surgery, Xijing Hospital of Digestive Diseases, Fourth Military Medical University, Xi’an, China; ^4^ Experiment Center for Science and Technology, Shanghai University of Traditional Chinese Medicine, Shanghai, China; ^5^ Department of Quality Testing & Research, Fuzhou Maixin Biotech Inc., Fuzhou, China

**Keywords:** microenvironment, proteomics, colon, stroma, adenoma, carcinoma

## Abstract

Tumor microenvironment (TME) is a key factor involved in cancer development and metastasis. In the TME of colorectal cancer (CRC), the gene expression status of stromal tissues could influence the CRC process from normal to adenoma then carcinoma; however, the expression status at the protein level has not yet been well evaluated. A total of 22 CRC patients were recruited for this study, and the tissue regions corresponding with adjacent, adenoma, and carcinoma were carefully excised by laser capture microdissection (LCM), including a patient with adenoma and carcinoma. The individual proteomes of this cohort were implemented by high-resolution mass spectrometer under data-independent acquisition (DIA) mode. A series of informatic analysis was employed to statistically seek the proteomic characteristics related with the stroma at different stages of CRC. The identified proteins in the colorectal stromal tissues were much less than and almost overlapped with that in the corresponding epithelial tissues; however, the patterns of protein abundance in the stroma were very distinct from those in the epithelium. Although qualitative and quantitative analysis delineated the epithelial proteins specifically typified in the adjacent, adenoma, and carcinoma, the informatics in the stroma led to another deduction that such proteomes were only divided into two patterns, adjacent- and adenoma/carcinoma-dependent. The comparable proteomes of colorectal adenoma and carcinoma were further confirmed by the bulk preparation- or individual LCM-proteomics. The biochemical features of the tumor stromal proteomes were characterized as enrichment of CD4+ and CD8+ T cells, upregulated pathways of antigen presentation, and enhancement of immune signal interactions. Finally, the features of lymphoid lineages in tumor stroma were verified by tissue microarray (TMA). Based on the proteomic evidence, a hypothesis was raised that in the colorectal tissue, the TME of adenoma and carcinoma were comparable, whereas the key elements driving an epithelium from benign to malignant were likely decided by the changes of genomic mutations or/and expression within it.

## Introduction

Colorectal cancer (CRC) is the second most common cancer worldwide, despite advances in diagnosis and treatment, including surgical resection with postoperative adjuvant chemotherapy, radiotherapy, and immunotherapy (1). Most CRC therapies focus on targeting to carcinogenic epithelium; however, it has become increasingly apparent that the survival and proliferative ability of carcinoma cells are largely determined by the tumor microenvironment (TME) (2–5). The TME, or tumor stroma, comprises the connective tissue and a variety of cell types, including lymph and blood vessels, inflammatory/immune cells, the extracellular matrix (ECM), and fibroblasts (6). Tumor stromal cells are thought to play an important role in CRC progression and drug resistance (7, 8). Therefore, it is thought that the tumor/stroma ratio could predict recurrence in CRC patients who were treated with neoadjuvant chemotherapy, as well as be a prognostic factor in stage II CRC (9). As the tumor stroma plays a pivotal role, the relevant mechanisms at the molecular level have been emerged to a mainstream in CRC research (8, 10–12).

A pronounced desmoplastic reaction in tumor stroma is an independent indicator of poor prognosis in CRC and its recurrence (13). Most commonly, under involvement of many regulators including TGF-β, PDGF, IL-4, IGF-II, and ECM proteins, stromal fibroblasts undergo transdifferentiation into cancer-associated fibroblasts (CAFs), which promotes tissue tension and tumor progression (14, 15). Infiltration of immune cells in response to CRC development is another important event in tumor stroma, while the types and quantities of immune cell infiltrated are assumed as vitally prognostic biomarkers for CRC. Downregulated Th1 cells with consequent events of cytokine reduction, such as IFN-γ, TNF-α, IL-12, and IL-18, are involved in the progression from adenoma to carcinoma in colorectal tissues, while a prominent response of either CD3+ T cell or antigen-specific cytotoxic T cells in stroma could improve the prognosis of CRC survival (16, 17). The information related to these molecules, which are common in signaling pathways and inflammatory regulation, is highlighted in studies on the CRC stroma, while an overall figure of molecular responses to CRC in the stroma is another important direction to explore the CRC molecular mechanism. The study based on high-throughput technology has been naturally deployed to dissect the high complexity of the CRC stroma. After surveying the CRC genomics data, Isella et al. emphasized the importance of assessing the stromal transcriptomic signals in a new CRC classification scheme, employing RNA-seq to discriminate stromal gene expression from cancer cells, and defining CRC subtype signature genes as having stromal or epithelial origin (2). More recently, using single-cell genome and transcriptome sequencing method in combination, Zhou et al. found that somatic copy number alterations were high in CRC tissues but most prevalent in immune cells, fibroblasts, and endothelial cells in both the tumor stroma and the normal tissues, while Zheng et al. revealed a high level of heterogeneity in CAFs and a nine-gene prognostic CAF-related signature in CRC stroma (18, 19).

The genome acts as a blueprint for gene expression; therefore, proteins play a critical role in the functioning of all living cells, and thus, proteomics of CRC stroma is an inevitable research topic. Over the last few decades, proteomic approaches have been employed to identify stroma-related proteins in response to CRC development and metastasis. Using target proteomics, Kahlert et al. performed a multiplex-based angiogenic cytokine and matrix metalloproteinases (MMPs) assay to quantify the expression of nine angiogenic cytokines and eight MMPs in tissue samples from CRC, obtained through laser capture microdissection (LCM), and found that stroma-derived proteins can act as prognostic biomarkers (20). Additionally, Sugai et al. conducted an immunohistochemistry (IHC) assay focusing on seven protein biomarkers specific to CAFs and the stroma and reported that the expression patterns of those biomarkers are useful for patient stratification into risk categories for lymph node metastasis in submucosal invasive CRC (21). Very recently, Wang et al. took a digital spatial profiler, a high-plex spatial proteogenomic technology, to analyze 40 antibodies with DNA oligomer conjugation and a panel of probes for 84 mRNAs related with epithelial and immune cells, and concluded that these specific biomarkers were differentially expressed between the tumor and stromal regions at either the transcriptional or translational levels (22). Freitas et al. analyzed the immune landscape of the colorectal adenomatous polyps using IHC and found that compared with low-grade dysplasia, high-grade dysplasia was characterized with decreased immune infiltration, increased MHC-I expression, and lower PD-L1 expression (23). With respect to profiling proteomics, LCM was a common means to isolate the tumor and adjacent stroma tissues, followed by proteomic analysis to identify the differentially expressed proteins (DEPs) between the tumor and adjacent stroma. Several stromal proteins, such as desmin and tenascin-C, have been identified as potential biomarkers of CRC (24, 25). In contrast to the advancing field of transcriptomics, relevant research on CRC-related stromal proteomics has proceeded at a slower pace.

While there is much room for improvement in proteomic investigations, large-scale proteomic surveys are challenging to conduct. Previous proteomics analyses have been restricted to a limited number of proteins that were responsive to the CRC stroma. Historically, target proteomics has only concentrated on well-known stromal proteins and lacks a broad enough view to discover novel candidates related to CRC stroma. Traditional proteomics, either isobaric tag labeling or two-dimensional electrophoresis, provided relative quantitative information but lack global and precise quantification necessary for exploring stromal DEPs. Another concern is the comparison of stromal proteomes. It is generally accepted that CRC develops from adenoma into carcinoma. In early reports, most investigators paid attention to the proteomic analysis onto the comparison of paired tissues, carcinoma and adjacent tissues, but rarely looked at the proteomic alternations of stroma in response to the development of adenoma and carcinoma. There has been an ambiguous scene of proteomes related to adjacent, adenoma, and carcinoma in colorectal tissues. With respect to individual proteomic differences, omics data are typically person-dependent; moreover, adenoma and carcinoma in colorectal tissues are reasoned more individual characteristic. An obvious barrier in proteomic data analysis is how to perform two different types of comparisons, adenoma versus carcinoma and person versus person, simultaneously. An appropriate solution to this issue is a well-designed sampling method for the collection of adenoma and carcinoma samples from the same patient and to implement data comparison for an individual as opposed to between individuals.

Based on these considerations, we initiated a proteomic project to survey stromal proteins responsive to adenoma and carcinoma of colorectal tissues. Most tissues of adjacent, adenoma, and carcinoma were individually sampled from CRC patients using LCM, after which tissue samples were subjected to proteomic analysis using high-resolution mass spectrometry and individual quantification in data-independent acquisition (DIA) mode. By integrating various datasets and bioinformatic algorithms, the analysis concluded that the epithelial proteomics were CRC stage-dependent, whereas the stromal proteomics in adenoma was comparable with that in carcinoma.

## Materials and Methods

### Collection of the Colorectal Tissues

**Figure 1:**
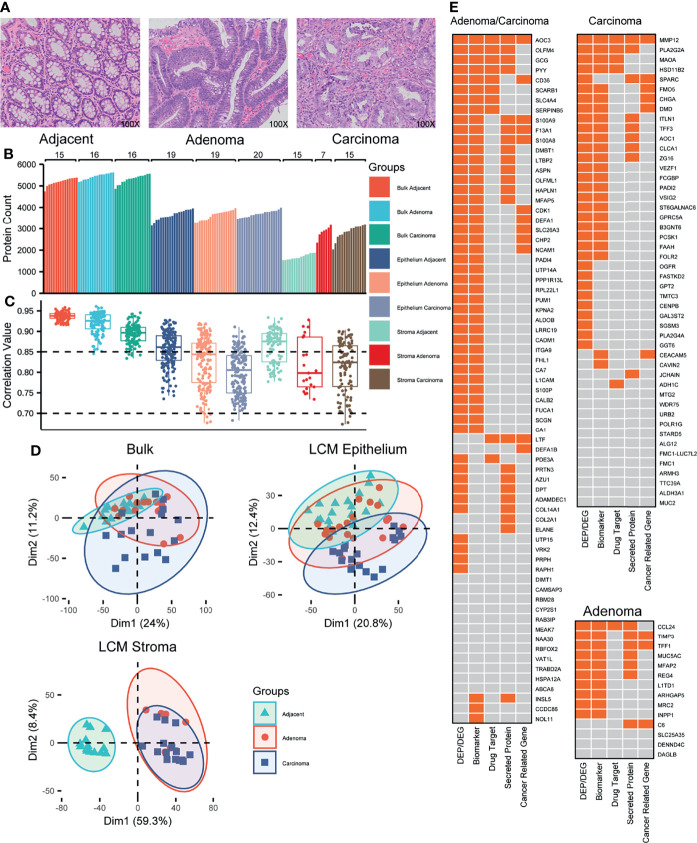
Proteomic overview of the colorectal tissues. **(A)** Representative images of colorectal tissues, including adjacent, adenoma, and carcinoma. Original magnification, ×100. **(B)** Distribution of the identified proteins in individual samples from 22 CRC patients. **(C)** Distribution of the protein abundance correlations among the samples within a group labeled. **(D)** PCA plots for the proteomes responsive to adjacent, adenoma, and carcinoma. **(E)** The biomarker heatmaps that recognize the adjacent from the adenoma and carcinoma (left), from the carcinoma (upper right), and from the adenoma (lower right) identified using both bulk and LCM samples. The boxes with orange indicate the CRC-related gene expression previously reported.

For bulk preparation, 47 colorectal tissues were surgically obtained from 19 treatment-naive CRC patients in Xijing Hospital, Xian, China. Samples included 15 adjacent tissues, 16 adenoma tissues, and 16 carcinoma tissues. Fresh tissue samples were divided into two parts, one delivered for pathologic examination and the other placed in liquid nitrogen at -196°C immediately until use. The tissues were stained with hematoxylin and eosin (H&E) and were assessed under a microscope by senior pathologists to diagnose the types and stages of colorectal diseases based on the tumor classification criteria of the World Health Organization (WHO) and AJCC 2017. Typical histopathology images for adjacent, adenoma, and carcinoma are depicted in **Figure 1A**. The detailed clinical parameters of patients are provided in **Table S1**. Informed consent was obtained from all patients involved in this study. The study protocol was approved by the Ethics Committee of the Xijing Hospital, Xian, China and the BGI Research Institute (NO. FT 15168).

For LCM preparation, colorectal tissues were collected from 22 treatment-naive CRC patients in the same hospital, including the patients who denoted tissues for bulk preparation. Samples were collected from the same hospitals mentioned above, after which tissue samples were further dissected to isolate tissues responsive to the stroma or epithelium using LCM, MMI Cellcut Plus (Molecular Machines & Industries, German). The tissues stored in liquid nitrogen were thawed, embedded in an optimal cutting temperature compound, and then refrozen in cryostat at -20°C. The refrozen tissues were sliced to 8-μm-thick sections, in which a tissue section was divided into two groups: indicator and target slides. Under MMI Cellcut Plus, the LCM was operated on the target slides with the guidance of indicator slides stained with H&E. A total of 95 LCM samples were obtained: 58 epithelial (19 adjacent, 19 adenoma, and 20 carcinoma) and 37 stromal tissues (15 adjacent, 7 adenoma, and 15 carcinoma). In these samples, five patients donated the complete set of tissues including the epithelium and stroma tissues at adjacent, adenoma, and carcinoma status. Several LCM-spotted areas from an LCM sample were pooled and stored at -80°C until use.

### Extraction of the Peptides Derived From the Colorectal Tissue Proteins

The tissues gained from either bulk or LCM preparation were suspended in the lysis buffer, 2% RapiGest, 100 mM NH_4_HCO_3_, 2 mM EDTA, and 1 mM PMSF, pH 8.0 followed by ultrasonication (VCX130, Sonics Inc, Newtown, CT, USA) and centrifugation at 20,000×g. The suspension was reduced with 10 mM DTT and was alkylated with 55 mM IAM, then digested with trypsin (1:50 μg/μg) in 100 mM NH_4_HCO_3_ at 37°C overnight. The resulting peptides were desalted using self-packed tip columns with OLIGO R3 reverse-phase resin. One-tenth of each sample was aliquoted, and all the aliquots from the digested samples were pooled for the construction of a peptide spectral library. Pooled samples were separated into ten fractions with self-packed tip columns using high-pH Xtimate C18 resin. The remaining volume of each sample was used for quantitative proteomic analysis.

### Quantitative Proteomics Using DIA

Identification and quantification of the peptides generated from the colorectal tissues were implemented under LC MS/MS system on Ultimate 3000 nanoLC coupled with a Q Exactive HF mass spectrometer (ThermoFisher Scientific, San Jose, CA, USA). To generate the spectrum library, the MS/MS signals were acquired in the data-dependent acquisition (DDA) mode at the following parameter settings: full-scan MS spectra (350–1,500 m/z) with a resolution of 120,000, HCD with 28% relative energy, and MS/MS scan at 15,000 resolution. To quantify the peptide quantities, MS/MS signals were obtained in the DIA mode at the following parameter settings: full-scan MS spectra (350–1,500 m/z) with a resolution of 120,000, HCD with 28% relative energy, fragmentation of precursor ions in sequential windows of 25 m/z, and MS/MS scan at 30,000 resolution. For global calibration of the retention time of peptides, iRT peptides (Ki-3002-1, BIOGNOSIS, Switzerland) were spiked into the digestive peptides at a ratio of 1:100. A peptide mixture was loaded onto a self-packed column (30 cm × 150 μm with 1.8 μm C18 resin) and eluted with a 120-min gradient. The eluted peptides were injected into the mass spectrometer through a nanoelectrospray ionization (ESI) interface.

### Peptide Analysis Based on MS/MS Signals

The MS/MS signals elicited from the DDA mode were input to a search engine, MaxQuant (v.1.6.0.1), and were searched against human SwissProt entries from the UniprotKB database (Uniprot, release 2018_02) with the following parameters: maximum missed cleavage at 1, fixed modification at cysteine carbamidomethylation, variable modification at methionine oxidation and N-terminal protein acetylation, and minimal peptide length at seven amino acids (26). The maximum false discovery rate (FDR) for peptide or protein identification was set at 0.01. The MaxQuant output was used to generate the spectral library for DIA analysis. The MS/MS signals acquired in the DIA mode were input into Spectronaut (v.12.4, BIOGNOSIS, Switzerland) for peptide and protein quantification using RT calibration by iRT. FDR was set both peptides and proteins less than 0.01.

### Tissue Microarrays and Immunohistochemistry (IHC) Staining

Commercial tissue microarrays (TMAs) (HLin-Dis060PT-01, Shanghai Outdo Biotech Co, China) were composed of 51 colorectal tissue samples, 7 normal tissues, 35 adenoma tissues, and 9 carcinoma tissues. Informed consent for these clinical samples was provided by the manufacturer. The antibodies used for IHC were gifted by MXB (Fuzhou Maixin Biotech. Co, China). CD4 (RMA-0620) and CD8 (RMA-0514) were used to represent signals of immune cells and VIMENTIN (MAB-0735) was used to represent signal of stromal cells. IHC staining was performed using MaxVivsion™ 3 HRP kits (KIT-5220, Fuzhou Maixin Biotech. Co, China), according to the manufacturer’s instructions; the staining results were screened using a pathological section scanner (Sunny Optical Technology, China). The stained images were deconvoluted using the IHC profiles plugin of the ImageJ software (version 1.53k, National Institutes of Health) (27). The stromal regions under a microscope were estimated by both images of the vimentin and H&E staining. To assess the intensity signals derived from CD4 or CD8, several positive spots in the stromal regions were randomly selected, and the total intensities of those spots were integrated to estimate the median intensity in each case. Comparisons of CD4 or CD8 staining intensity under different circumstances were statistically evaluated in all cases in the tissue microarrays.

### Statistical Analysis of Proteomic Data

All statistical analyses except the prediction of immune cell fractions from LCM samples and gene set enrichment analysis (GSEA) were performed in the R open-source statistical programming software. Protein abundances estimated from individual samples were globally normalized and transformed to log format using the MSstats package (28). Imputation of missing values for proteins with less than 50% frequency of missing detection was conducted using the pca function in the pcaMethods (29) package. The resulting principal component analysis (PCA) was applied to evaluate differences in protein expression among sample groups using the factoextra package, and a weighted correlation network analysis (WGCNA) was performed to assess the protein clusters in response to tumors using the WGCNA package (30). Stromal cell infiltration was estimated using the ESTIMATE package (31). Overrepresentation analysis of KEGG terms among DEPs was performed using the ClusterProfiler package (32). Hierarchical cluster analysis was performed using the pheatmap package. Immune cell fractions from LCM samples were predicted using CIBERSORTx (http://cibersortx.stanford.edu), with a signature matrix generated from Rieckmann’s proteomics dataset, including nine major immune cell types (33, 34). GSEA was run in the GSEA software (35). Ligand–receptor interaction was analyzed using the iTALK package (36). Except for the plots mentioned above, all plots were constructed in ggplot2, ggpubr, circlize, and ggalluvial packages in R (37, 38).

## Results

### Proteomic Atlas of the CRC and Its Adjacent Tissues Gained by Bulk or LCM Preparation

On average, 5,324 proteins were identified per sample of the bulk-prepared colorectal tissues (5,200 in adjacent, 5,447 in adenoma, and 5,323 in the carcinoma region), whereas 3,667 and 2,471 proteins were identified in epithelial and stromal samples with LCM preparation, respectively (**Figure 1B**). Compared to the epithelium, there were significantly fewer proteins detected implying that the stroma contained more structural tissues and fewer protein components. In the LCM preparation for epithelial tissue, 3,651 proteins in adjacent, 3,641 in adenoma, and 3,710 in carcinoma regions were identified, while for stromal tissue, 1,697 in adjacent, 2,866 in adenoma, and 2,851 in carcinoma regions were found, respectively. Regarding the overlapping proteins of bulk and LCM preparations in the same samples, most of the proteins from LCM were also identified in the bulk-prepared samples, for instance, 89.8% in LCM adjacent epithelium, 91.1% in LCM adenoma epithelium, and 90.1% in LCM carcinoma epithelium (**Figure S1**).

The correlation of protein abundance between any random paired samples (Spearman’s correlation) could reflect the homogeneous status of samples in a group. **Figure 1C** shows the correlation between the abundance correlation of identified proteins within one of the sample groups. The correlation coefficients of all the sample groups, either bulk or LCM, were above 0.7, revealing a moderate or higher abundance correlation for the identified proteins within a group sample. In bulk preparation, the correlation medians among the three groups, carcinoma, adenoma, and adjacent, were all above 0.85; the median from the adjacent samples was the highest, while the median from the carcinoma samples was the lowest, indicating that the heterogeneity of protein expression was extended in the abnormal tissues, adenoma, and carcinoma (**Figure 1C**, left). In LCM preparation, the correlation coefficients of all the groups were generally spread as compared to those of the bulk samples (**Figure 1C**, middle and right). It was an understandable observation because the samples obtained from LCM preparation came from pooling of several LCM slices, which might truly represent the heterogeneity status of protein abundance distribution within microtissues. As similar as bulk preparation, the correlation medians in the LCM adjacent regions were obviously higher than that of the LCM abnormal regions. The evidence for the protein heterogeneity changes in abundance responding to the sample preparations from bulk or LCM, which strengthens the argument that protein abundance is potentially correlated with colorectal tissues from carcinoma to adenoma to their microenvironment.

To determine whether the samples in different groups exhibited different specific protein characteristics, the quantitative proteomes were analyzed using a PCA in an unsupervised mode. In bulk preparation, there were several key components that caused considerable segregation between carcinoma and adjacent samples; however, there was a lack of such components to discriminate the adenoma samples from the carcinoma or adjacent samples (**Figure 1D**, upper left), implying that the adenoma might contain the transition characterization of protein abundance from adjacent to carcinoma. In LCM preparation, the PCA plot revealed that in epithelium, some protein features in carcinoma were distinguishable from those in adjacent or adenoma tissues; however, few features were distinguishable between adjacent and adenoma (**Figure 1D**, upper right). The conclusion derived from the upper left of **Figure 1D** was in general agreement with that from the upper right of **Figure 1D**. Moreover, in the stroma, the key components of protein abundance in adjacent were so specific as to differ adjacent from adenoma and carcinoma, whereas the two proteomes of the latter were highly overlapped (**Figure 1D**, lower left). The PCA onto the epithelial and stromal proteomes thus led a deduction that the adenoma epithelium is in the progress of normal to malignant so that its proteomic status across possesses the features of adjacent and carcinoma, while the similarity of stroma proteomes in both adenoma and carcinoma hints a comparable microenvironment in colorectal tumors.

Which enlightenment to guide further study was derived from the generally proteomic data? The qualitative scale of identified proteins in the stroma of both adenoma and carcinoma was obviously larger than that in adjacent (**Figure 1B**), while the correlation medians in the adjacent stroma regions were apparently higher than those in the stroma of both adenoma and carcinoma (**Figure 1C**). The qualitative proteomics unveiled similar extent of protein expression in the stroma of adenoma and carcinoma, which was distinct from that in the adjacent regions, while the diverse status of stroma proteomes among individual adenoma or carcinoma samples partially reflected the enhanced heterogeneity of the microenvironment in CRC patients. The unsupervised PCA on the quantification of overlapped proteins in three stromal tissues, adenoma, carcinoma, and adjacent, resulted in that the key protein components of adenoma stroma were clustered with those of carcinoma, whereas they were segregated from those in adjacent (**Figure 1D**), suggesting the basically biochemical features shared by adenoma and carcinoma stroma.

Identifying DEPs responsive to tumors is a key step in identifying CRC-related biomarkers. With greater than a four-fold change in abundance and a q-value less than 0.05, six sets of DEPs generated from bulk and LCM preparations were acquired and are listed in **Table S2**. The numbers of overlapping DEPs between bulk and LCM in epithelium were 208 for carcinoma vs adjacent (C/N), 88 for adenoma vs adjacent (A/N), and 67 for carcinoma vs adenoma (C/A). In the co-identified proteins by both bulk and LCM, there were more DEPs found in the LCM samples versus the bulk samples (**Figure S2**), further supporting the idea that the proteomics of the LCM samples for carcinoma might truly reflect the heterogeneity status. In total, 363 overlapped DEPs were taken into WGCNA, and the statistical evaluation of WGCNA resulted in three modules, carcinoma-, adenoma-, and carcinoma/adenoma-related, each with node significance in both datasets (**Figure S3**). As certain proteins in the same module were common between bulk and LCM preparations, further filtration to remove the shared proteins based on the WGCNA modules resulted in 132 DEPs, in which the carcinoma-, adenoma-, and carcinoma/adenoma-related DEPs were 48, 14, and 70, respectively. The biological and oncological significance of these DEPs was determined through published references and HPA datasets, as illustrated in **Figure 1E**. Of these DEPs, other studies reported 70% (93 DEPs) as the colorectal abnormalities at the protein or mRNA level, 16% (21 DEPs) were categorized as the pan-cancer genes (such as LTF, S100A9, and CDK1), 30% (39 DEPs) were identified as secreted proteins (most of which were likely found in the cytoplasm and potentially detectable in body fluid), and 12% (16 DEPs) were regarded as the drug targets. In summary, these results support those in previous studies, which necessitates further exploration of novel CRC biomarkers.

### Proteomic Characteristic of Colorectal Epithelium and Stroma

**Figure 2:**
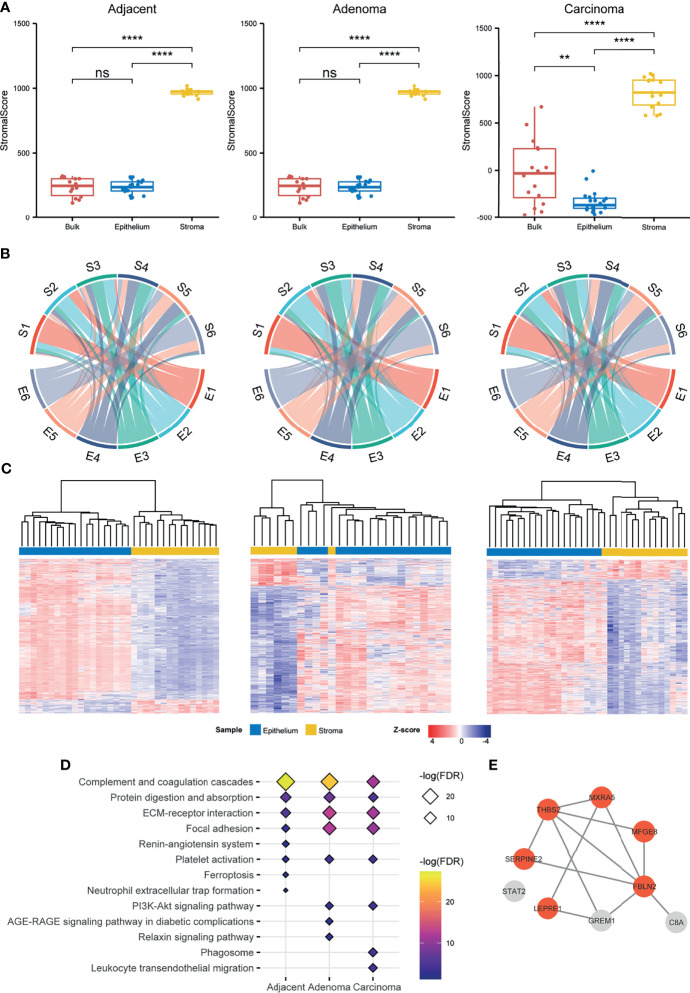
Comparison of the stromal proteomes to the epithelial ones in the adjacent, adenoma, and carcinoma tissues. **(A)** Evaluation of the infiltration level of stromal cells in a preparation by ESTIMATE based on proteomic data. The signs of ** and **** represent a significance with p < 0.01 and p < 0.0001, respectively, and ns represents nonsignificance. **(B)** Comparison of the proteomic abundance patterns between stroma and epithelium in three different circumstances using chord plots. Each proteome, stroma (S) or epithelium (E) is evenly divided into six patterns according to the abundance order. **(C)** Hierarchical clustering for the DEPs between stroma and epithelium in three different circumstances. The normalized abundance is represented by Z-scores. **(D)** Enrichment of the KEGG pathways for the upregulated DEPs and unique proteins of stroma in three different circumstances. The enriched pathways ranked above top 8 are listed in the plot. **(E)** The STRING network of nine stromal biomarkers selected through a stringent filtration. The circles with orange indicate fibroblast specific proteins.

ESTIMATE, which was developed by Kosuke et al. to infer the fraction of stromal cells in a cell admixture based on gene expression, was employed to estimate the stromal scores of protein abundance acquired from this study and to predict the infiltration level of stromal cells in the preparations. As shown in **Figure 2A**, the stromal scores in the stroma preparation were significantly higher than those in the epithelium and bulk preparation, regardless of the adjacent, adenoma, or carcinoma categorization. The stromal samples prepared by LCM were representative of the specific features of stromal proteins, indicating that the stromal cells were heavily infiltrated. Closely looking at the distribution of stromal scores in the bulk and epithelial samples, the diverse degree in bulk appeared larger than that in epithelium because of bulk with partial stroma. The ESTIMATE evaluation provided evidence that the proteomic features of the stroma were clearly distinct from those of epithelium, both adenoma and carcinoma.

A comparison of the proteins identified in the epithelial and stromal regions across all tissue types (adjacent, adenoma, and carcinoma) is exemplified in **Figure S4**, revealing that most identified proteins in the stroma were co-detected in the epithelium regardless of tissue type with 96%, 91%, and 92% of stroma proteins overlapping with that of the epithelium of adjacent, adenoma, and tumor, respectively. Generally, the sizes of identified proteins in the epithelium samples across all tissue types were comparable, whereas those in the adjacent stroma were obviously smaller than those found in the stroma of adenoma and carcinoma. A question was raised regarding the abundance patterns of shared proteins in the stroma, and if they were similar to those in the epithelium. To clarify the issue, the shared proteins were simply ranked by mean values of abundances in each tissue and then equally divided into six groups from high to low protein abundance, defined as E1–E6 for epithelium and S1–S6 for stroma (**Figure S5**). Then, a circular plot was drawn to better visualize the differences in abundance orders in the stroma and epithelium of all three tissue types, as shown in **Figure 2B**. In the two tissues, an average of 74.7% of the higher abundance proteins ranked at E1 was similar to that in S1, while 68.9% in E6 comparable with that in S6 in all types of tissues (**Table S3**), indicating that in each tissue type, the proteins in the two groups with higher or lower abundance in stroma were comparable to those in the epithelium. In contrast, the ranked proteins in the other groups appeared to have a large diversity in epithelium and stroma. In the adjacent samples, an average of about 33.8% of the ranked proteins in stroma, S2 to S5, remained in an abundance order similar to that in epithelium, E2 to E5; in the adenoma samples, approximately 48.2% in stroma versus in epithelium; and in the carcinoma samples, approximately 41.7% in stroma versus in epithelium. Regarding the proteins with middle abundance, a large percentile of them in stroma exhibited converse changes in abundance as compared with that in epithelium under all the circumstances. Even though over 90% of the stroma proteins were overlapped with the epithelial proteins, the fact in **Figure 2B** depicts that the stromal proteins with own specific abundance patterns might play different roles in functions from the epithelial ones. The circular plot was also employed in individually sorting protein abundance patterns of paired epithelial and stromal samples and resulted in similar conclusions as in **Figure 2B**, which further supports the specific abundance characteristics of the shared proteins in epithelium and stroma (**Figure S6**).

To mine the shared proteins of stroma and epithelium with significant differences in abundance, the DEPs were defined by setting the parameters of q-value < 0.05 and fold change > 2, resulting in a total of 1,486, 882, and 1,280 shared DEPs between stroma and epithelium recognized in adjacent, adenoma, and carcinoma, respectively. Hierarchical clustering with these DEPs against three circumstances is depicted in **Figure 2C**, demonstrating that the DEPs of the stroma and the epithelium can be distinguished by their abundance. The abundance patterns were summarized in **Table S4**, revealing that the overlap rate of the DEPs among adjacent, adenoma, and carcinoma was relatively low about 14.6%, and the majority of overlapped DEPs in epithelium held relatively higher abundance compared with that in stroma. As regards the DEPs whose abundance in stroma was significantly higher than that in epithelial and the stromal unique proteins, a total of 437 such proteins in carcinoma were defined, 383 in adenoma, and 222 in adjacent, respectively. These stromal proteins were further categorized through the prediction of functional enrichment in the KEGG database using criteria at FDR < 0.05. **Figure 2D** displays the eight most enriched pathways in each tissue type in which 62.5% of pathways were shared across tissue types. The shared pathways with higher protein counts are enriched in the functions of focal adhesion, ECM–receptor interaction, protein digestion and absorption, and complement-related pathways, which is consistent with current knowledge of stromal functions.

The identified proteins in stroma were highly overlapped with those in epithelium regardless of the circumstance, adenoma, carcinoma, or adjacent. Did the overlapped proteins in stroma exhibit their characteristic against those in epithelium? As illustrated above, the specific features of stromal proteins were appraised in three angles, evaluation of stromal scores using ESTIMATE (**Figure 2A**), comparison of abundance patterns for the shared proteins, and DEPs between stroma and epithelium in the three circumstances (**Figures 2B, C**). All the assessments directed a conclusion that the proteomic traits owned to stroma were very discriminated from the proteomic features of epithelium in all the circumstances.

The protein biomarkers of colorectal tumors in the stroma were further evaluated using the following criteria: 1) uniquely identified in stroma regardless of tissue type, 2) co-identified in over 80% of the patients in the cohort, 3) DEPs of stroma in adenoma and/or carcinoma, and 4) abundance ranked within 80% of total proteins with more than two peptides. In total, nine biomarkers were defined in the carcinoma stroma, including FBLN2, MFGE8, THBS2, SERPINE2, MXRA5, STAT2, C8A, P3H1, GREM1, and PTGDS, which was only one in the adenoma stroma, respectively. Of the nine biomarkers of carcinoma stroma, eight were predicted using the STRING database as an interaction network, including six fibroblast specific proteins as defined by Human Protein Atlas (HPA) (**Figure 2E**). Importantly, all six fibroblast proteins were reported as CRC-related; therefore, this protein panel would be regarded as the CRC indicators in the stromal region. In a future study, all the specific antibodies against these stromal proteins will be generated for IHC examination at a large scale using tissue microarray, and based upon an IHC survey, more indicative and specific antibodies are selected for clinical diagnosis. Taking all the evidence above at different angles, stromal biomarkers, abundance rank, DEPs, enriched pathways, and CRC biomarkers of stroma, the epithelium- and stroma-dependent patterns elicited from proteomes were established from the colorectal tissues collected by LCM in the individual CRC patients.

### Comparison of the Stromal Proteomes Derived From Colorectal Adenoma and Carcinoma

**Figure 3:**
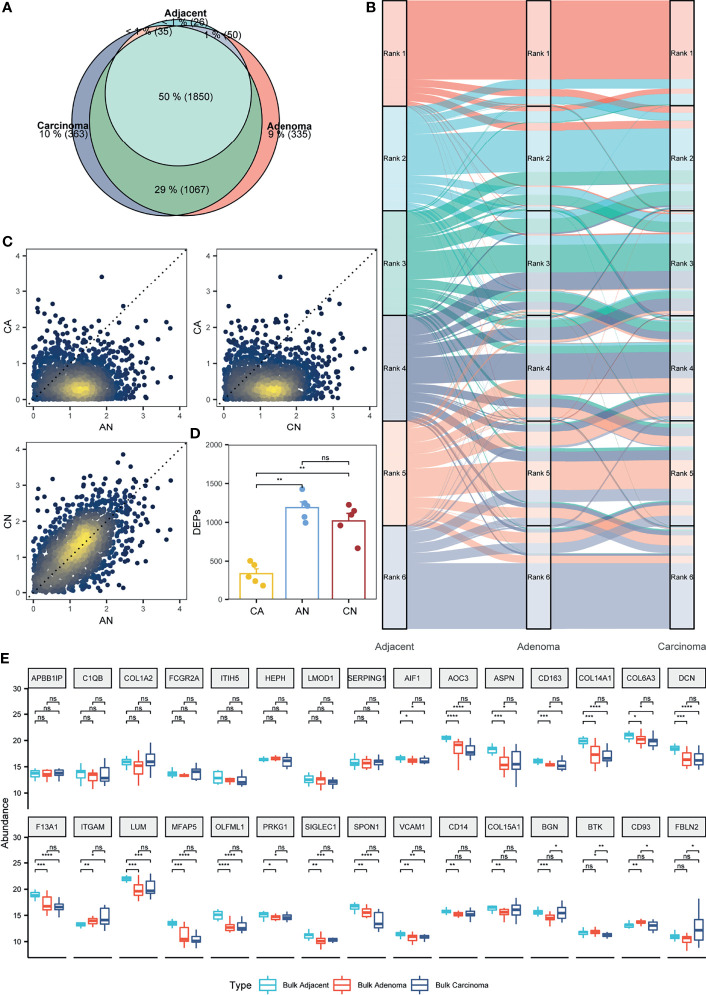
Appraisal of the stromal proteomes in the adjacent, adenoma, and carcinoma tissues. **(A)** Overlap analysis to the stromal proteins identified in the adjacent, adenoma, and carcinoma tissues. **(B)** Comparison of the abundance patterns of stromal proteomes among the adjacent, adenoma, and carcinoma tissues using the alluvium plot. Each stromal proteome in a tissue is ranked at six abundance groups. **(C)** Comparison of the abundance ratios of stromal proteins that were generated by the division of the abundance of overlapped proteins in a tissue to that in another tissue, such as adenoma versus adjacent (AN), carcinoma versus adenoma (CA), and carcinoma versus adjacent (CN). **(D)** Statistical evaluation toward the stromal DEPs of AN, CA, and CN using Wilcoxon test. The signs of ** and ns represent a significance with p < 0.01 and nonsignificance. **(E)** Statistical evaluation toward the stromal biomarkers of ESTIMATE in the three bulk tissues, adjacent, adenoma, and carcinoma using Wilcoxon test. The signs of *, **, ***, and **** represent a significance with p < 0.05, p < 0.01, p < 0.001, and p < 0.0001, respectively, and ns, represents nonsignificance.

In contrast to epithelium, the sizes of the identified proteins in stroma from adjacent, adenoma, and carcinoma were quite flexible, with ones in adjacent significantly lower than those in the other tissues (**Figure 1B**). Furthermore, looking at the overlap status of the stroma proteins (**Figure 3A**), most proteins identified in adjacent stroma (94.3%) were co-identified in adenoma and carcinoma; however, they only occupied 55.9% and 56.0% of the proteins in the stroma of adenoma and carcinoma, respectively. The stromal proteins identified in adenoma tissue highly overlapped with those in carcinoma (78.8%), whereas the unique proteins in each tissue (about 10%) exhibited a low abundance level (**Figure S7**). The qualitative information led to an intimation that the molecular functions of the adjacent stroma were different from those in adenoma and carcinoma, while the functions in the two latter tissues were similar. As shown in **Figure 1D**, the PCA, combined with qualitative and quantitative proteomics, demonstrated a visible separation of principle features in the adjacent stroma from that in the adenoma or carcinoma; nevertheless, the information deducted from **Figure 3A** shows little segregation ability between the adenoma and carcinoma stroma.

Focusing on quantitative proteomics, an alluvial plot was used to delineate the differences of abundance orders among the proteins co-identified in all the three stromal tissues. The plot reveals that the order of protein abundance in adjacent is distinct from adenoma or carcinoma, whereas those in adenoma and carcinoma are comparable (**Figure 3B**). The alluvial plot focusing on the shared proteins between adenoma and carcinoma also showed little change in the order of protein abundance distribution between adenoma versus carcinoma tissues (**Figure S8**). Moreover, the abundance ratios of the shared proteins in two tissues, a set of typical parameters to indicate functional diversity, were loaded to evaluate the comparability among the stromal samples. As depicted in **Figure 3C**, the ratios of adenoma/adjacent (AN) and carcinoma/adjacent (CN) are around the diagonal, whereas those of AN versus carcinoma/adenoma (CA) or CN versus CA are biased from the diagonal, implying that the distribution of protein abundance in adenoma is similar to carcinoma, but different from adjacent tissues. In addition, the DEPs between adjacent and adenoma or adjacent and carcinoma were 987 and 1,147, respectively; however, those between adenoma and carcinoma were only seven, indicating that there were few stromal proteins that can be distinguished between adenoma and carcinoma. Collectively, all quantitative comparisons in protein abundance provided evidence of comparable proteomes between adenoma and carcinoma.

In this study, paired samples from five CRC patients of adjacent, adenoma, and carcinoma tissues were collected and subjected to proteomic analysis. Therefore, the diversity and similarity of tissue proteomes can be assessed at the individual level. For each CRC patient, the abundance ratios of the share stromal proteins from CA, AN, or CN are approximated and presented in **Figure S9**. In all the five individuals, the abundance ratios of the stromal proteins of CN or AN are around the diagonal, whereas those of CA are biased from the diagonal, which supports the conclusions drawn from **Figure 3C** at the individual proteomics level. The DEPs of CA, CN, or AN for all individuals were defined based on the criteria of fold change ≥ 2, and the distribution of those DEPs in different circumstances was statistically assessed, as shown in **Figure 3D**. The DEP scales in the five CRC individuals in CN are not significantly different from those in AN (approximately 1,104 DEPs); nevertheless, the DEPs defined in CN or AN are significantly higher than those in CA, which are only at the 332 DEP level. Additionally, the DEPs of CN overlapped with 63.8% of AN, whereas the DEP overlap of CA with that of CN or AN was only 7.3% (**Table S5**). The protein abundance and DEP analysis at the individual level also endorsed the argument of which the proteomes of the adenoma stroma were comparable with those of the carcinoma stroma.

As the bulk CRC tissues contained both epithelial and stromal cells, the abundance distribution of the specific stroma proteins was assumed predictable based on the bulk proteomes. A total of 141 stromal protein biomarkers were listed in ESTIMATE, of which 30 proteins identified in the bulk-prepared samples were found in the list. These proteins were selected for the evaluation of their abundance distribution in the bulk tissues of adjacent, adenoma, and carcinoma. **Figure 3E** shows a statistical comparison of protein abundance among the three colorectal tissue types, indicating that 26 proteins remained a consistent abundance in the adenoma and carcinoma tissues, and only four proteins in adenoma had the significant changes in abundance compared with that in carcinoma. Of the 30 stromal markers, 18 proteins with comparable abundance in adenoma and carcinoma were very different from those in adjacent. Therefore, the bulk proteomics was likely to reach a similar conclusion, in which the proteomic characteristics of adenoma stroma were basically equal to those of carcinoma stroma.

Since the stromal proteins in the adjacent regions were very distinct from those in adenoma and carcinoma, while the two latter proteomes were comparable, for the first time, the observation based on proteomic evidence was headed for a hypothesis that the microenvironment around adenoma was close to that around carcinoma, and the pathological development of the two tumors were determined by lesion cells themselves.

### Microenvironmental Characteristic Shared by Colorectal Adenoma and Carcinoma

As shown above, the stromal proteome exhibited the specific characteristic in which both qualification and quantification of the proteomes derived from colorectal adenoma and carcinoma appeared similar. The two sets of stromal proteomes thus could be merged into one, simply termed as CAC. A question was naturally raised, what are the basic biochemical features of CAC? It is well known that the immune response of TME is a critical element to block or to facilitate tumor cell growth. The functional characteristic of the CAC dataset therefore was primarily converged to three immune-related properties, such as immune cells, antigen presentation, and ligand–receptor communication.

**Figure 4:**
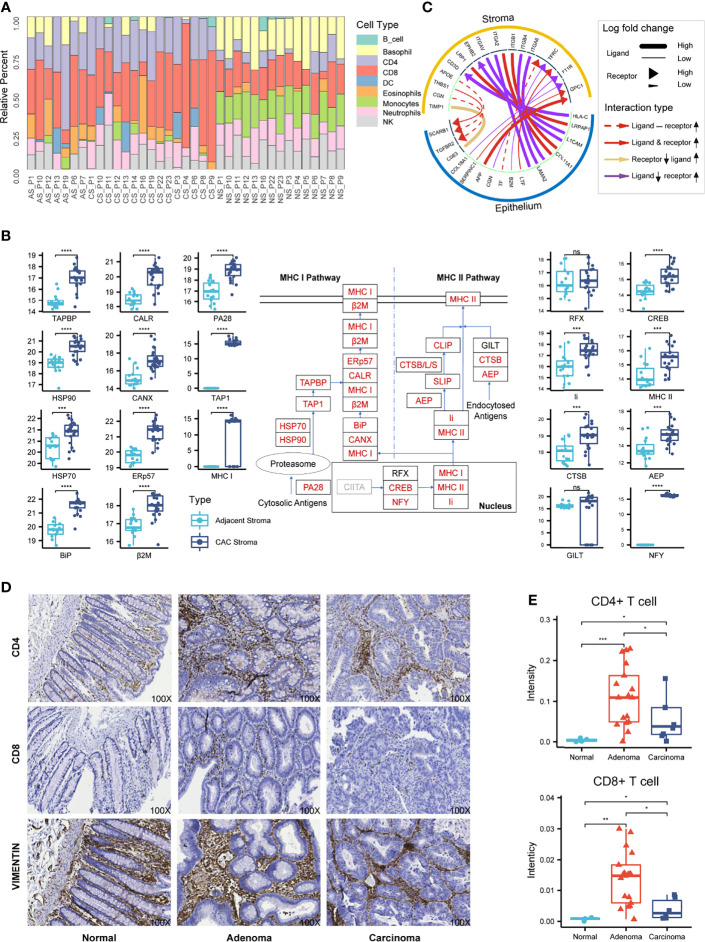
Characterization of the stromal proteomes in the adjacent, adenoma, and carcinoma tissues. **(A)** Distribution of immune cell infiltration in the stromal tissues of adjacent, adenoma, and carcinoma based on analysis of CIRBERSORTx to the stromal proteomes. The prefix of sample names in the x-axis is the abbreviation of tissue type: AS, CS, and NS represent adjacent stroma, adenoma stroma, and carcinoma stroma, respectively. **(B)** Statistical evaluation to the abundance of the stromal proteins participating in the antigen presentation pathway between the adjacent and CAC tissues. The diagram of this pathway is placed at the center, in which the rectangles with red stand for the upregulated proteins, with black for no significant change and with gray for unidentified. The boxplots of abundance comparison are depicted on the left for the proteins in MHC I and on the right for those in MHC II and the nucleus. The signs of *, **, ***, and **** represent a significance with p < 0.05, p < 0.01, p < 0.001, and p < 0.0001, respectively, and ns, represents nonsignificance. **(C)** The typical ligand–receptor interactions between the stromal and epithelial proteins predicted by iTALK. **(D)** The representative IHC images using CD4, CD8, and vimentin antibody on the tissue microarray of CRC. Original magnification, ×100. **(E)** Statistical comparison of the CD4 or CD8 staining intensities among the normal, adenoma, and carcinoma stromal tissues in the tissue microarray of CRC. The signs of *, **, and *** represent a significance with p < 0.05, p < 0.01, and p < 0.001, respectively.

CIBERSORTx, an informatic tool to typify cell composition in a complex tissue based on gene expression data, was used to analyze the matrix of immune-responsive proteins derived from CAC. The CIBERSORTx analysis resulted in the immune cell fractions in two stromal tissues, adjacent and CAC, as shown in **Figure 4A**, in which nine distinct immune cell types were found based on the proteomics data. Although the immune cell compositions varied from patient to patient, typical differences in the cell compositions between adjacent and CAC were evident. In most adjacent stroma, monocytes and natural killer (NK) cells occupied relatively higher composition, whereas in most CAC stroma, CD4+, CD8+, and DCs had relatively larger contents. This came to a deduction of which in the stroma of both adenoma and carcinoma, the tumor suppression was strengthened due to enrichment of CD4+ and CD8+ T cells, and their regulatory cells, DC, whereas the tumor surveillance was weakened as NK cell antitumor immunity and its regulatory, monocytes, were reduced in the tumor microenvironment site. The deduction was also endorsed by the enriched biomarkers of CD4+ and CD8+ T cells in the stroma of adenoma and carcinoma. By checking such biomarkers in the individual patients who donated the colorectal tissues from adjacent, adenoma, and carcinoma (**Figure S10**), the types or quantities of those CD4+ and CD8+ T-cell biomarkers in the stroma of adenoma and carcinoma were obviously larger than those in the adjacent.

Gene Set Enrichment Analysis (GSEA), an algorithm that focuses on the functional responses from groups of genes that share common properties within a cell, was used to evaluate pathway enrichment among stromal and epithelial samples. The GSEA prediction derived from the proteome datasets showed that antigen processing and presentation in CAC was ranked at one of the top enriched pathways, even though it was inactivated in either adenoma or carcinoma epithelium (**Figure S11**). Generally, the antigen presentation pathway in a tumor microenvironment is enhanced followed by the activation of immune cells, especially CD4+ and CD8+ T cells. The abundance augment of the proteins participating in antigen presentation pathway thus supported the deduction derived from the CIBERSORTx analysis above. In terms of protein abundance in the antigen presentation pathway, 19 of the 22 proteins are significantly upregulated in CAC, either MHC I or MHC II pathway, which provides strong evidence of the activation state of this pathway in CAC (**Figure 4B**).

The method of iTALK for characterizing and illustrating intercellular communication signals in a multicellular system was employed to survey the intercellular communication between tumor cells and the microenvironment. The iTALK analysis of the proteome datasets of the stromal and epithelial samples revealed significant changes in ligand–receptor pairs from adjacent to tumor (**Figure 4C**). In total, 195 ligand–receptor pairs exhibited change in abundance in response to tumor. After the removal of the redundant ligands or receptors that interacted with different ligand/receptors of epithelium, the 55 proteins in stromal sides remained, of which 46 functioned in immune infiltration, antigen presenting, immune suppression, and other immune regulation functions. The phenomenon that over 80% upregulated ligand–receptor interactions in stroma were involved in immune responses offered another evidence of immune enhancement in the CRC microenvironment.

The result of the comparable microenvironment shared by adenoma and carcinoma based on proteomic evidence was further verified by an alternative approach of IHC on the tissue microarray of CRC. Antibodies against CD4 and CD8 were selected as the immune responsive signals and that against vimentin was taken as the specific antigen of colorectal stroma. The typical IHC images are presented in **Figure 4D**, which did not reveal the stronger staining signals of immune responses in the stromal regions but not in the epithelial regions (all the images of tissue microarray depicted in **Figure S12**). To quantitatively assess the staining intensities in response to CD4 and CD8 in the stroma, the combined images of H&E staining and vimentin IHC image were used to define stromal regions, while several IHC positive spots for CD4 or CD8 within a stromal region were randomly picked and integrated for the estimation of the intensity median. The intensity medians of CD4 and CD8 from seven normal tissues, 35 adenoma tissues, and 9 carcinoma tissues are statistically summarized in **Figure 4E**, which indicates that the CD4 and CD8 staining intensities in adenoma and carcinoma were significantly higher than those in normal, whereas the two immune-responsive signals in adenoma were much higher than those in carcinoma. The IHC images provided supportive evidence for the proteomic deduction of higher CD4+ and CD8+ T-cell infiltration in the microenvironment of adenoma and carcinoma. On the other hand, as the IHC images were obtained by the specific antibodies with different titers while the CIBERSORTx profiles were extracted from the proteomic data, the quantitative conclusions to enriched CD4+ and CD8+ T cells elicited from the two approaches appeared diverse somehow.

## Discussion

By surveying the literatures regarding CRC investigation, especially using proteomic approaches, this study chose a new angle to focus on the proteomic responses of colorectal stroma to the CRC development, from adenoma to carcinoma. Specifically, the relevant experiments were carefully designed at three aspects: 1) to monitor the proteomic changes related to adjacent, adenoma, and carcinoma tissues, with emphasis on the tissue samples from one patient with both adenomas and carcinomas; 2) to excise the stromal or epithelial tissues using LCM to ensure that the microtissues had viable representative characteristics; and 3) to individually profile the proteomes of microtissues with DIA using a high-resolution mass spectrometer aimed at high-quality data of protein identification and quantification, from either bulk or LCM preparation. In general, this study produced novel information, including the result that the proteomes of the adjacent stroma were significantly different from those of the tumor stroma; however, the two stromal proteomes of the adenoma and carcinoma were comparable, indicating that the TME was relatively consistent in colorectal tissues. In addition, the total stromal proteins identified were much less than that perceived in the epithelium of both adjacent and tumor tissues and were mostly overlapped by the epithelial proteins; nevertheless, the stromal proteins in majority held own abundance features that were very different from the epithelium shared ones. Finally, with a stringent criterion, a panel consisting of nine proteins was selected as stromal biomarkers of CRC. Quantitative proteomics based upon LCM preparation of CRC tissues, therefore, provides a new perspective to understand the microenvironment of CRC.

Although few studies have specifically focused on the stromal proteome related to colorectal adenoma, some studies have targeted proteins in the stromal regions of carcinoma. Two reports aimed at quantitatively comparing the stromal proteomes in CRC patients using LCM and iTRAQ-based MS quantification and claiming several DEPs (25, 39). Compared with the current DIA MS, the isobaric tag technology is restricted by identification and quantification of proteins in large size, while DEPs with a small scale were unlikely to be statistically enriched in certain pathways for mechanism exploration. As shown in **Figure 1B**, over 3,000 proteins were identified and quantified in the stromal tissues in this study, which is the largest scale study in stromal proteomics so far. The proteins identified by iTRAQ were almost completely covered by the DIA data. The high quality of stromal proteomics with more identified proteins and multiple individual proteomes of the CRC patients formed a foundation upon which to identify the proteomic features of the microenvironment of adenoma and carcinoma. Large sizes of the CRC-related gene expression have been reported by several transcriptomic studies based on the RNAseq or single-cell sequencing techniques. Other studies focused on the transcriptomic characteristic of adenoma stroma, nevertheless, are of several investigations to specifically mention the DEGs between adjacent and carcinoma stroma. Whether the pathways of immune responses are upregulated in carcinoma stroma remains debatable. Shen et al. defined the transcriptomic modules of adjacent and carcinoma stroma and asserted the immune-related pathways enriched in normal stroma but not in carcinoma stroma (40), whereas Zhang et al. found that immune cells, such as macrophages and conventional DCs, were accumulated in the microenvironment of CRC (40, 41). More recently, integrative analysis of single-cell transcriptomic and image atlas toward 62 CRC patients revealed that there were two most common human colorectal polyps, conventional adenomas and serrated polyps, in which the former arisen from WNT-driven expansion of stem cells, while the latter were derived from differentiated cells through gastric metaplasia (42). That study proposed that a cytotoxic immune microenvironment partially related with antigen-presentation differences might precede hypermutation and tumor cell-differentiation status. The deduction elicited from the proteomic survey herein supports the hypothesis of which the cell differentiation in colorectal polyps is decided by the types of adenoma cells and is facilitated by TME.

Cells of the lymphoid lineage in the CRC TME demonstrate dual function, the first of which is tumor suppression *via* production of pro-inflammatory cytokines or direct tumor cell killing, and conversely, second, supporting tumor progression *via* angiogenesis and invasion by favoring cell proliferation. Correlations between the infiltration of immune cells and disease prognosis have been widely reported in the study of CRC TME. For example, Toor et al. investigated lymphoid populations within the CRC TME of 50 CRC patients (43). The levels of CD4+ and CD8+ T cells in TME were found significantly higher than those in the CRC tumor, while the patients with mismatch-repair deficiency/microsatellite instability were accompanied with high levels of CD4+ and CD8+ T cells. There are two generally acceptable mechanisms of immune escape at least: lack of CD8+ T-cell infiltration and CD8+ T-cell dysfunction. Uddin et al. identified numerous significantly upregulated DEGs in CRC stroma and brought a deduction that CD8+ T cells were more enriched in colon tumor stroma than normal stroma (44), while Di et al. applied single-cell mass cytometry to mold the T-cell phenotype in 18 CRC patients and revealed increased CD8+ in CRC TME (44, 45). Involvement of lymphoid cells in CRC stroma, however, is not in general agreement in the research frontier. Using flow cytometry for a comprehensive analysis of immune cells in tumor tissues from a cohort of 69 CRC patients, Li et al. claimed that no significant difference was found for the CD4+ and CD8+ T cells between tumor and normal tissues (46), while Freitas et al. examined the immune context of sporadic and familial adenomatous polyposis lesions along the colorectal adenoma–carcinoma sequence and observed an overall decrease in tumor-infiltrating immune cells along the colorectal tumors (23). During CRC development, immunoediting leads to the generation of plentiful neoantigens presented by both MHC-I and MHC-II that basically require synergetic effects of CD4+ and CD8+ T cells. Whether the pathways of neoantigen presentation through MHC are coordinated with CD4+ and/or CD8+ in CRC is not clearly clarified yet. It is worth noting that the approaches for the investigations regarding lymphoid lineage of CRC TME so far often relied on transcriptional measurements or IHC staining with limited biomarkers. The study described herein made another attempt to monitor the changes of functional molecules in CRC TME, protein profiling toward the stromal regions related to adenoma and carcinoma. The enrichment of CD4+ and CD8+ T cells in CAC was reinforced by both evidence of the CIBERSORTx analysis based on the stromal proteomes and IHC onto tissue microarray. The pathway status of antigen presentation in response to CAC was inspected through all the participants of MHC pathways and possible immune interactions. More importantly, the alterations of immune reaction of either T cells or pathways were derived from the statistical assessment to the cohort and were verified in all the individual cases whose had the stromal materials of adenoma and carcinoma. The proteomic evidence, for the first time, lays a foundation of the infiltration of immune lineages and the upregulation of antigen presentation pathways in the TME of CRC.

## Data Availability Statement

The original contributions presented in the study are included in the article/**Supplementary Material**. Further inquiries can be directed to the corresponding authors.

## Ethics Statement

The studies involving human participants were reviewed and approved by Institutional Review Board on Bioethics and Biosafety of BGI (NO. FT 15168). The patients/participants provided their written informed consent to participate in this study.

## Author Contributions

SL and QZ conceived this study and prepared the manuscript. KY designed the experiments, analyzed the data, and prepared manuscript. BB designed the clinical sampling and collected the samples. YR guided the experimental procedure. XZ, HZ, and YL assisted the data analysis. LC and JZ assisted the experiment design. All authors approved the final version of the manuscript.

## Conflict of Interest

Authors KY, YR, XZ, HZ, YL, LC, JZ and SL are employed by Beijing Genomics Institution. Authors BC and QY were employed by Fuzhou Maixin Biotech Inc.

The remaining authors declare that the research was conducted in the absence of any commercial or financial relationships that could be construed as a potential conflict of interest.

## Publisher’s Note

All claims expressed in this article are solely those of the authors and do not necessarily represent those of their affiliated organizations, or those of the publisher, the editors and the reviewers. Any product that may be evaluated in this article, or claim that may be made by its manufacturer, is not guaranteed or endorsed by the publisher.

## Abbreviations

CAC, colorectal adenoma and carcinoma; CAF, cancer-associated fibroblast; CD3, cluster of differentiation 3; CD4, cluster of differentiation 4; CD8, cluster of differentiation 8; CIBERSORT, cell type identification by estimating relative subsets of RNA transcripts; CRC, colorectal cancer; DC, dendritic cells; DEP, differentially expressed protein; DDA, data-dependent acquisition; DIA, data-independent acquisition; ECM, extracellular matrix; FDR, false discovery rate; GSEA, Gene Set Enrichment Analysis; H&E, hematoxylin and eosin; IHC, immunohistochemical; IL, interleukin; KEGG, Kyoto Encyclopedia of Genes and Genomes; LCM, laser capture microdissection; MMP, matrix metalloproteinase; N/A/C, normal/adenoma/carcinoma; NK, natural killer; PCA, principal component analysis; TME, tumor microenvironment; WGCNA, weighted correlation network analysis; WHO, World Health Organization.
