# Supplementary material for: The Comparable Microenvironment Shared by Colorectal Adenoma and Carcinoma: An Evidence of Stromal Proteomics
Source: Front Oncol. 2022 Apr 1;12:848782. doi: 10.3389/fonc.2022.848782 (PMC9010820; doi:10.3389/fonc.2022.848782)
Supplement: Supplementary file 1 [file DataSheet_1.pdf]

The Comparable Microenvironment Shared by Colorectal Adenoma and Carcinoma: An Evidence of Proteomics

Keqiang Yan[1,2†], Bin Bai[3†], Yan Ren[2,4], Benliang Cheng[5], Xia Zhang[1,2], Haichao Zhou[1,2], Yuting Liang[1,2], Lingyun Chen[1,2], Jin Zi[2], QinghaiYang[5], Qingchuan Zhao[3*], Siqi Liu[1,2*]

Contents

Table S1. Clinical characteristics of colorectal cancer patients

Table S2. DEPs number identified in both LCM epithelium and bulk samples

Table S3. Protein overlap percentage between epithelium and stroma in each rank group

Table S4. DEP and stromal specific protein number identified in each tissue

Table S5. DEPs shared by CA and CN or AN comparison's groups in five individuals

Figure S1. Comparison of protein identification results.

Figure S2. The overlap of differential expression proteins (DEPs) in each group.

Figure S3. Workflow of epithelial biomarker selection.

Figure S4. Comparison of protein identification results between stroma and epithelium in each stage.

Figure S5. Proteins were divided into six groups based on rank of protein abundance.

Figure S6. Protein expression patterns between stromal and epithelial samples in five individuals.

Figure S7. Distribution of adenoma or carcinoma specific proteins in each group.

Figure S8. Protein patterns comparison between stromal adenoma and carcinoma

Figure S9. Fold change distribution between two comparison groups.

Figure S10. Abundance comparison of CD4 and CD8 related proteins.

Figure S11. GSEA results in three groups.

Figure S12. IHC results of tissue microarray stained with CD4, CD8 and vimentin.

Table S1. Clinical characteristics of colorectal cancer patients

	Total (n=22)
Age, mean±SD	59.6 (14.6)
BMI, mean±SD	23.6 (3.4)
Gender	
Male	18 (81.8%)
Female	4 (18.2%)
KRAS	
Mutate	11 (50.0%)
Wide Type	9 (40.9%)
NA	2 (9.1%)
Location	
Ascending Colon	2 (9.1%)
Ileocecum	2 (9.1%)
Sigmoid Colon	6 (27.3%)
Transverse Colon	1 (4.5%)
Rectum	11 (50.0%)
TNM stage	
I	5 (22.7%)
II	10 (45.5%)
III	5 (22.7%)
NA	2 (9.1%)

Notes: Categorical variables are presented as percentage. Continuous variables are presented as mean±SD.

Table S2. DEPs number identified in both LCM epithelium and bulk samples

Comparison Groups	LCM Epithelium		Bulk Tissue	
	Up-regulation	Down-regulation	Up-regulation	Down-regulation
Carcinoma/Adjacent	469	427	488	331
Adenoma/Adjacent	289	282	434	208
Carcinoma/Adenoma	329	288	210	267

Table S3. Protein overlap percentage between epithelium and stroma in each rank group

Overlap Percentage	E1-S1	E2-S2	E3-S3	E4-S4	E5-S5	E6-S6
Adjacent Overlap	67%	39.1%	32.4%	29.5%	34.3%	62.2%
Adenoma Overlap	80.1%	57.3%	42.5%	42.9%	49.9%	73.5%
Carcinoma Overlap	76.9%	47.5%	37.3%	37.5%	44.6%	71%

Table S4. DEP and stromal specific protein number identified in each tissue

Groups	DEPs	Up-regulation	Down-regulation	Stromal specific
Adjacent	1486	1353	133	89
Adenoma	882	726	156	227
Carcinoma	1280	1119	161	276

Table S5. DEPs shared by CA and CN or AN comparison's groups in five individuals

UNIPROT ID	SYMBOL ID	Patient Coverage
O75312	ZPR1	2
P01833	PIGR	2
P02788	LTF	3
P02792	FTL	2
P04179	SOD2	2
P04839	CYBB	2
P05362	ICAM1	2
P06703	S100A6	2
P07108	DBI	2
P07996	THBS1	2
P08134	RHOC	2
P08246	ELANE	2
P10809	HSPD1	2
P13284	IFI30	2
P13611	VCAN	3
P14678	SNRPB	3
P18084	ITGB5	2
P31689	DNAJA1	2
P31947	SFN	2
P40306	PSMB10	2
P47897	QARS1	2
P50454	SERPINH1	3
P55769	SNU13	2
P56537	EIF6	2
P61020	RAB5B	2
P61086	UBE2K	2
P61604	HSPE1	2
P78406	RAE1	2
Q02790	FKBP4	2
Q08380	LGALS3BP	3
Q14562	DHX8	2
Q15063	POSTN	2
Q99715	COL12A1	3
Q9ULC5	ACSL5	2
Q9Y6Q5	AP1M2	2

Notes: Proteins with patient coverage more than one are shown in table.

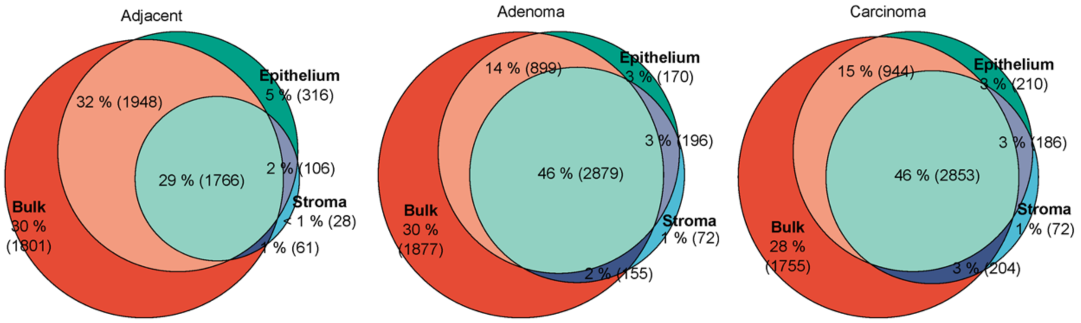

Figure S1. Venn diagram of proteins in each tissue.

Protein identification results in stroma, epithelium and bulk were compared in adjacent (left), adenoma (center) and carcinoma (right), respectively.

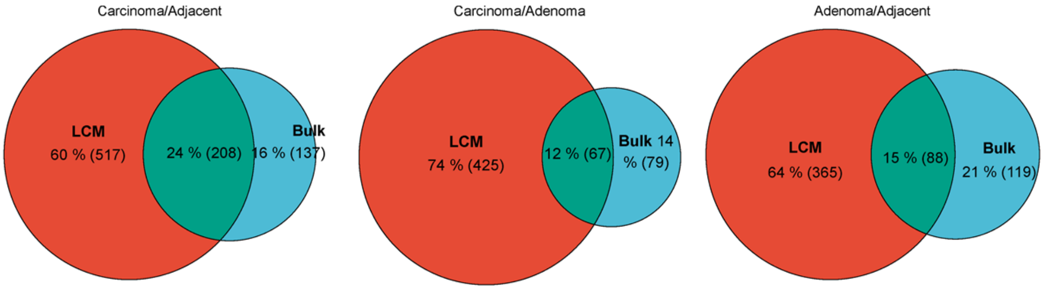

Figure S2. Venn diagram of differential expression proteins (DEPs) in each comparison group.

The panel compared DEPs identified in bulk and LCM samples at three comparison groups, carcinoma/adjacent (left), carcinoma/adenoma (center) and adenoma/adjacent (right). LCM samples were colored in red, bulk samples were colored in blue and overlaps between bulk and LCM samples were colored in green.

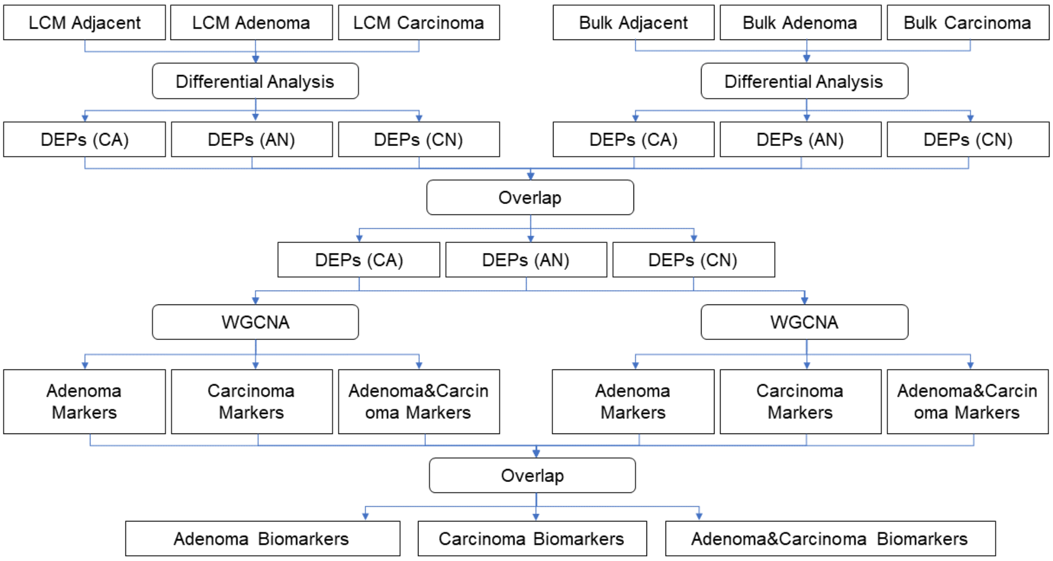

Figure S3. Analysis workflow of epithelial biomarker selection.

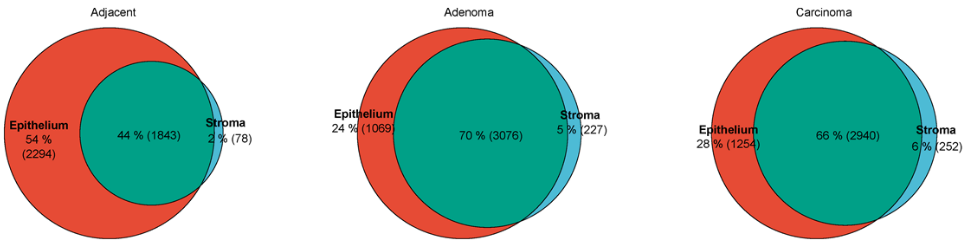

Figure S4. Venn diagram of protein identification results between stroma and epithelium.

Protein comparison between epithelium and stroma in three different stages, epithelial specific part was colored in red, stromal specific part was colored in blue, overlap part was colored in green.

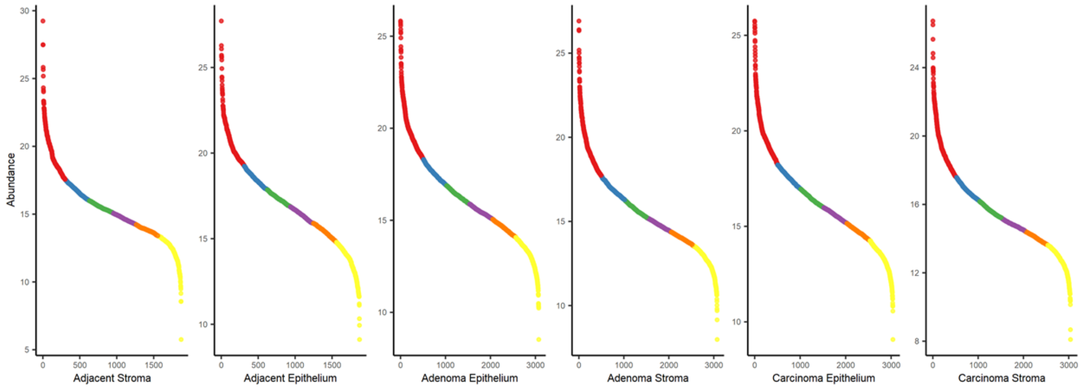

Figure S5. Protein abundance rank curve in each sample.

Proteins in each sample were divided into six groups based on rank of protein abundance. Each color represents different abundance range in each sample.

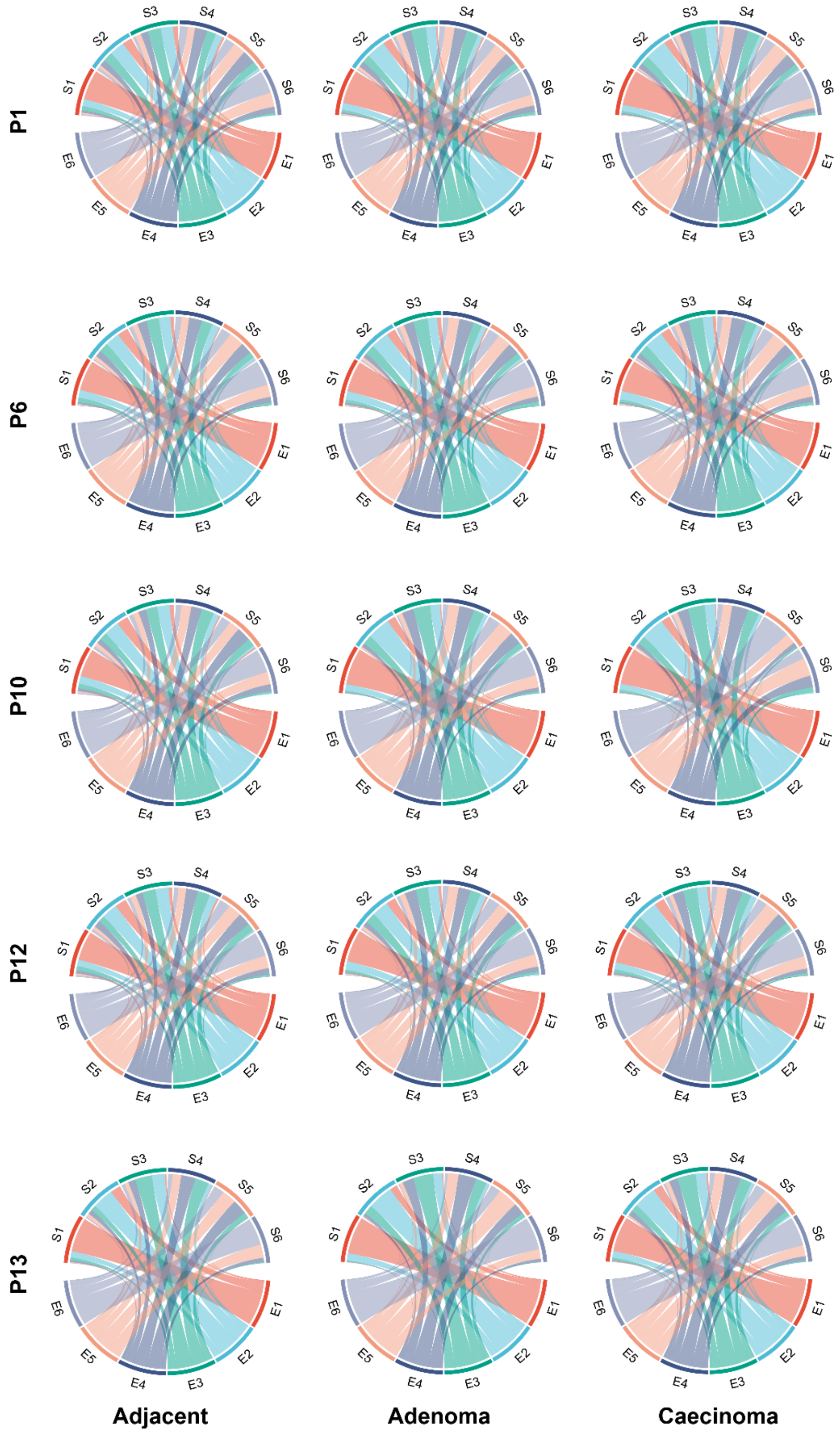

Figure S6. Protein expression patterns between stromal and epithelial samples in five individuals.

S1-S6 represent proteins in stroma, with abundance decreased from S1 to S6. E1-E6 represent proteins in epithelium, with abundance decreased from E1 to E6. The upper panel represents protein expression pattern comparison in adjacent, the middle panel represents expression pattern comparison in adenoma and the lower panel represents expression pattern comparison in carcinoma.

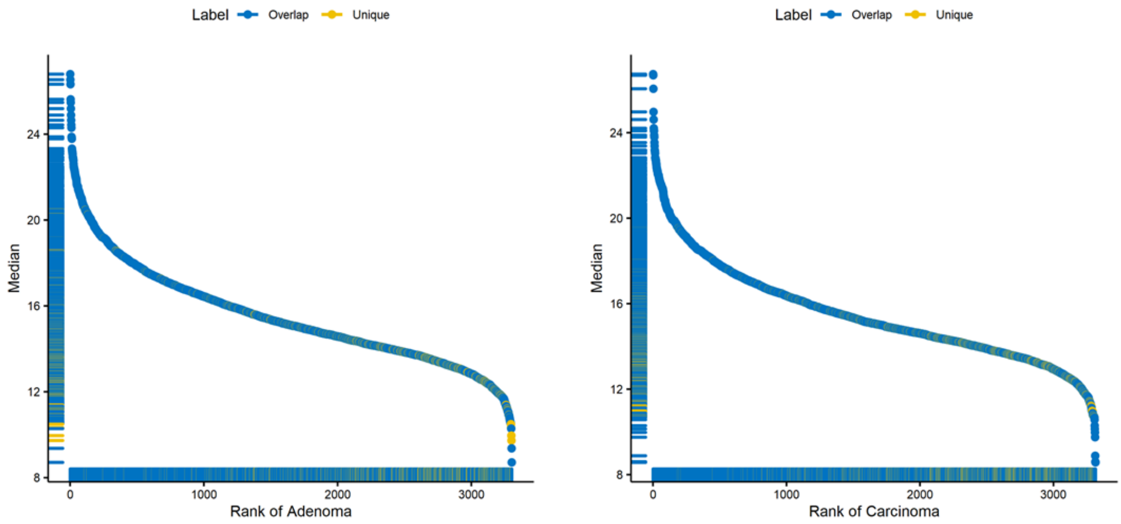

Figure S7. Distribution of adenoma or carcinoma specific proteins in each group.

Left figure represents protein abundance rank distribution in adenoma, while the right figure represents protein abundance rank distribution in carcinoma. Specific proteins in each group were colored in yellow, while proteins shared between adenoma and carcinoma were colored in blue.

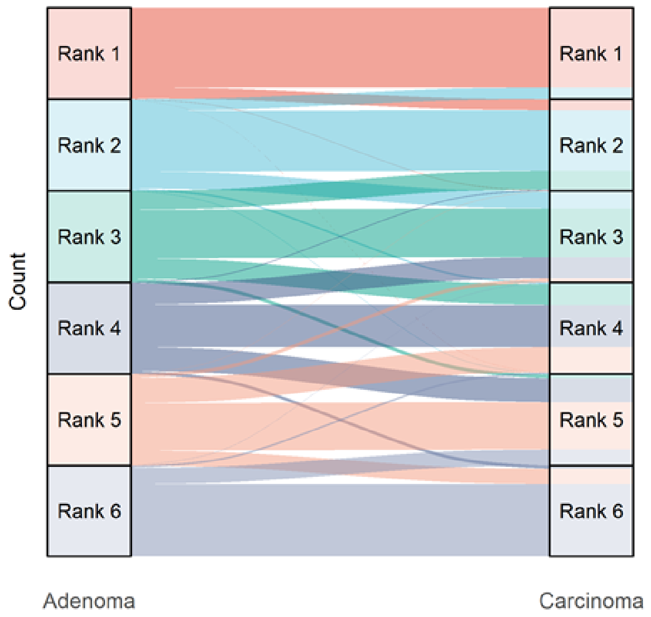

Figure S8. Protein patterns comparison between stromal adenoma and carcinoma.

Proteins shared between adenoma and carcinoma were divided into six rank groups based on their abundance in adenoma samples, with Rank 1 to Rank 6 represent protein abundance rank in decreased order.

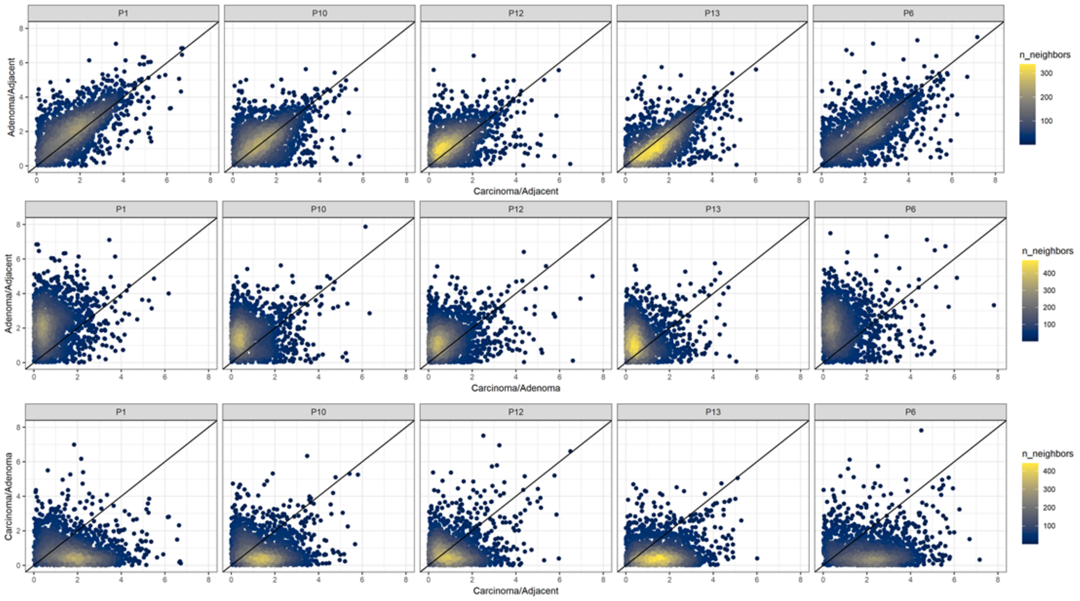

Figure S9. Scatter plot comparing abundance ratios between two comparison groups.

Abundance ratios between comparison of carcinoma/adjacent (x-axis) and adenoma/adjacent (y-axis) were in upper panel, comparison of carcinoma/adenoma (x-axis) and adenoma/adjacent (y-axis) were in middle panel, comparison of carcinoma/adjacent (x-axis) and carcinoma/adenoma (y-axis) were in lower panel. Points density was represented by color.

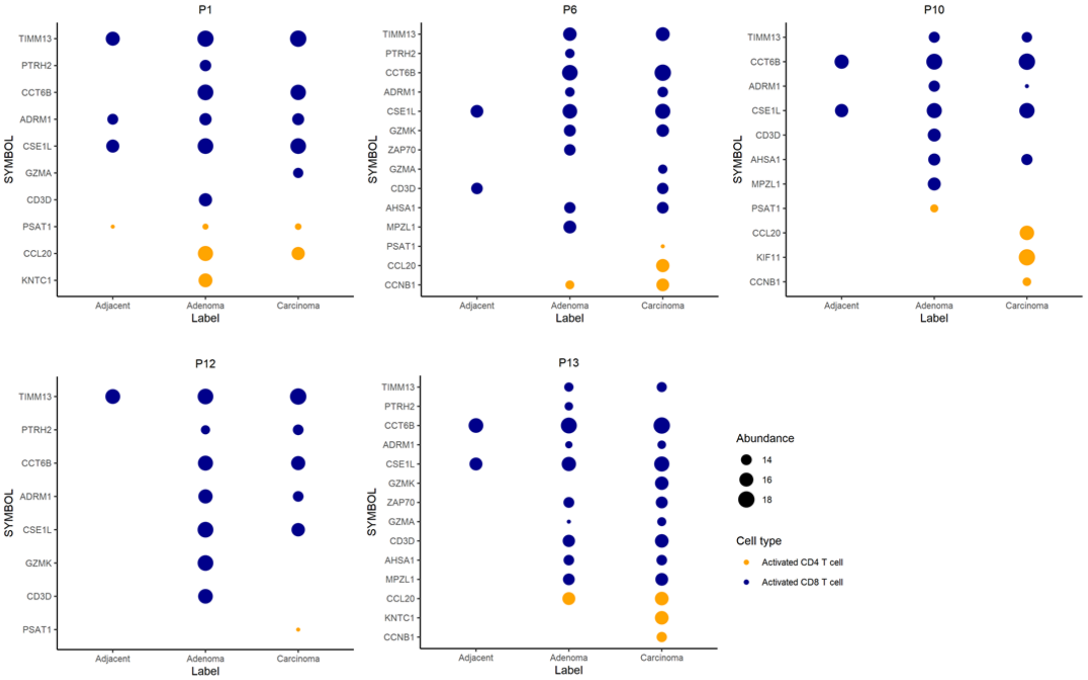

Figure S10. Abundance comparison of CD4 and CD8 related proteins.

Abundance of CD4 and CD8 related proteins were compared in three groups, adjacent, adenoma and carcinoma. Proteins of each cell type were denoted by colored.

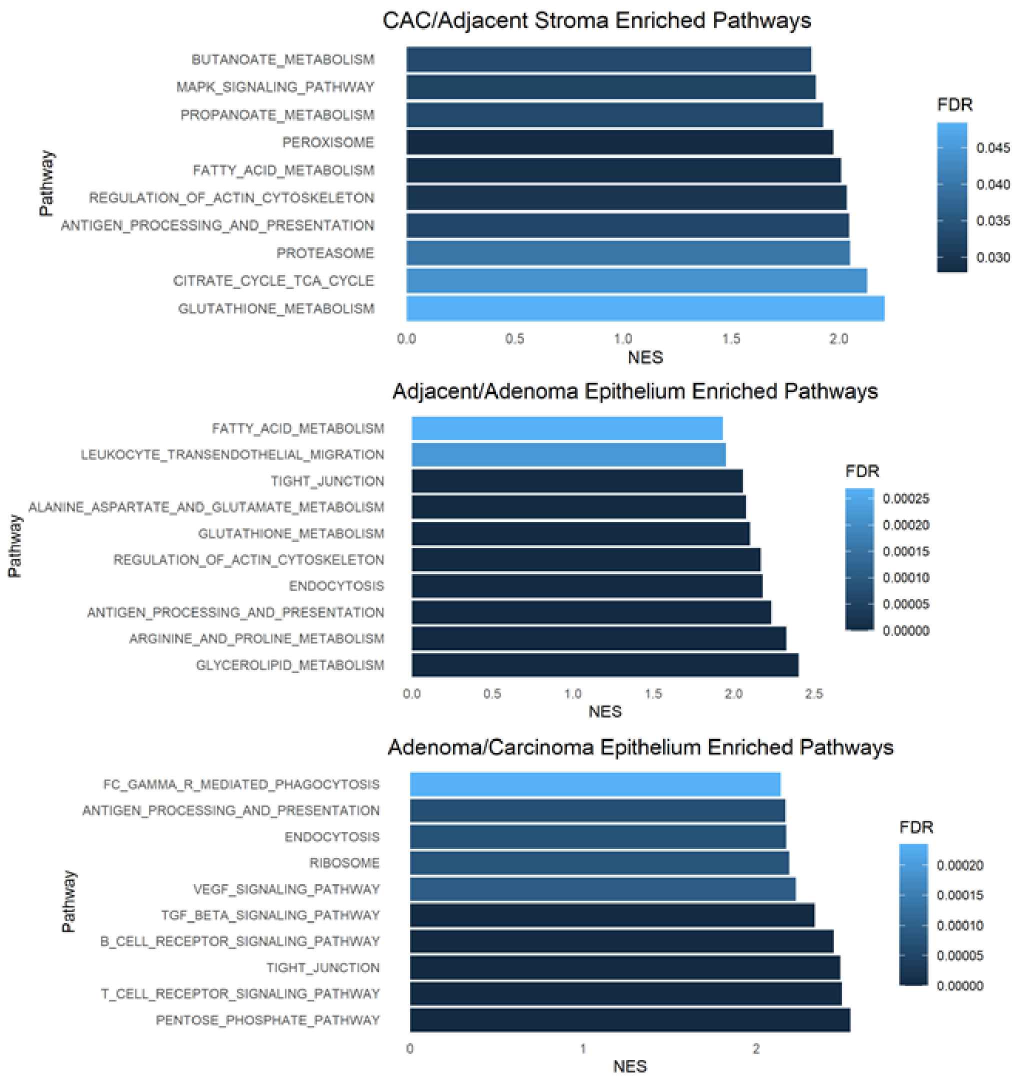

Figure S11. GSEA results in three comparison groups.

Pathway terms enriched by GSEA in groups of CAC stroma/adjacent stroma (upper figure), adjacent epithelium/adenoma epithelium (middle figure) and adenoma epithelium/carcinoma epithelium (lower figure). The x-axis represents NES score of each term, the y-axis represents enriched pathway terms and the color bar represents FDR.

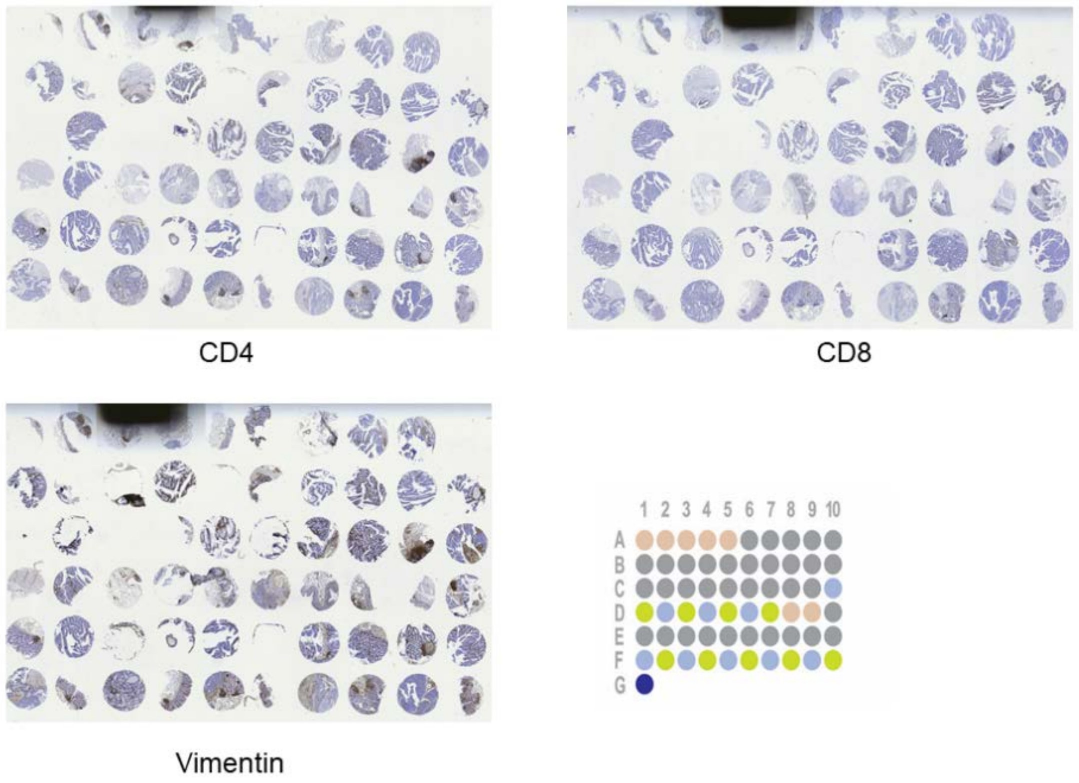

Figure S12. IHC results of tissue microarray stained with CD4, CD8 and vimentin.
The lower right picture represents tissue type in each point position. Normal colon samples were labeled in orange, adenoma samples labeled in grey, carcinoma samples labeled in blue and paired adjacent samples labeled in green. The darkblue point represents blank. Sample images that were contaminated during IHC experiment (showed in black area) were not used for statistic analysis.
